# Review of Chemistry for Students

**Published:** 1851-05

**Authors:** 


### Part 2—Reviews and Notices of New Works.

ARTICLE I.

*Review of Chemistry for Students, adapted to the courses as taught in the principal Medical Schools of the United States.* By John G. Murphy, M. D.; pp. 328, 12 mo.; Lindsay & Blakiston, Philadelphia, 1851, (from the publishers.)

This is an exceedingly well arranged and convenient little book. It gives the most important facts and principles of chemistry in a clear and very concise manner ; so as to subserve the object for which it is designed most admirably. It is not intended for a text book, but is to refresh the memory of those who have passed through a thorough course of the study of the science. The principle objection to such books, is, that they are liable to take the place of the more elaborate works, and thus make superficial scholars of students ; but it is very clear that one must first have a very good knowledge of chemistry, or this book will not be well enough understood to make it interesting—and hence, it will soon be laid aside. But to one who has been well taught previously, it will be a means of bringing very rapidly and clearly in review before his mind all of the more important points in his course of study, in which case it may be exceedingly useful. It is a fact well understood, that chemistry requires frequent refreshing in the mind to prevent its loss, even by those who, when students, had made the greatest proficiency in it, and the systematic works upon the subject require more time for their perusal than can in many instances, be devoted to it, consistently with the active duties of the practitioner, in which cases we should think the little book before us admirably adapted to supply their place.

It is printed in the publishers usual good style, and illustrated by a large number of diagrams.

